# Prestroke statins use reduces oxidized low density lipoprotein levels and improves clinical outcomes in patients with atrial fibrillation related acute ischemic stroke

**DOI:** 10.1186/s12883-019-1463-7

**Published:** 2019-10-18

**Authors:** Lanying He, Ronghua Xu, Jian Wang, Lili Zhang, Lijuan Zhang, Wang Zhao, Weiwei Dong

**Affiliations:** 1 Department of Neurology, The Second People’s Hospital of Chengdu, Chengdu, 610021 People’s Republic of China; 2 Department of Neurosurgery, The Second People’s Hospital of Chengdu, Chengdu, 610021 People’s Republic of China; 3 Department of Neurology, The Second Affiliated Hospital of Chengdu College, Nuclear Industry 416 Hospital, Chengdu, 610021 People’s Republic of China; 4 Department of Neurology, Yongchuan Hospital, Chongqing Medical University, Chongqing, China 610020 People’s Republic of China; 5 Department of Neurology, First Affiliated Hospital, Chongqing Medical University, Chongqing, China 400030 People’s Republic of China

**Keywords:** Acute ischemic stroke, Atrial fibrillation, Outcome, Statins, Oxidized low density

## Abstract

**Background:**

Atrial fibrillation (AF) is a common cause of cerebral infarction, which could lead to endothelial dysfunction, increased reactive oxygen species (ROS) and oxidized low density lipoprotein (Ox-LDL).AF is associated with higher mortality and more severe neurologic disability. Statins may exert neuroprotective effects that are independent of LDL-C lowering. The purpose of our study was to investigate whether prestroke statins use could reduce plasma Ox-LDL levels and improve clinical outcomes in patients with AF-related acute ischemic stroke (AIS).

**Methods:**

This was a multicenter prospective study that involved four medical centers, 242 AIS patients with AF were identified, who underwent a comprehensive clinical investigation and a 72 h-Holter electrocardiogram monitoring. All patients were divided into two groups: prestroke statins use and no prestroke statins use groups, who were followed up for 3 months. Plasma Ox-LDL levels were measured using enzyme-linked immunosorbent assay (ELISA) on admission and at 3 months. The outcome was death, major disability (modified Rankin Scale score ≥ 3), and composite outcome (death/major disability) at 3 months after AIS.

**Results:**

One hundred thirty-six patients were in no prestroke statins use group, and 106 in prestroke statins use group. Plasma Ox-LDL levels were significantly lower in prestroke statins use than in no prestroke statins use on admission and at 3 months (*P* < 0.001). Plasma Ox-LDL levels on admission were associated with 3-month mortality [adjusted odds ratio (OR), 1.05; 95% confidence interval (CI), 0.99–1.12; *P* = 0.047]. In fully adjusted models, prestroke statins use was associated with reduced 3-month mortality [adjusted OR, 0.38; 95% CI, 0.16–0.91; *P* = 0.031)], major disability (adjusted OR, 0.38; 95% CI, 0.15–0.99; P = 0.047), and composite outcome (adjusted OR, 0.31; 95% CI, 0.17–0.74; *P* = 0.009).

**Conclusions:**

Prestroke statins use can reduce plasma Ox-LDL levels and improve clinical outcomes in patients with AF-related AIS.

## Background

Ischemic stroke is the leading cause of death and disability among Chinese adults. About 13–26% of all acute ischaemic strokes are related to non-valvular atrial fibrillation (AF), the most common cardiac arrhythmia globally [1, 2]. It is believed that prethrombotic forces, along with endothelial dysfunction and blood stasis of AF are the basis of thrombus formation and growth, when large thrombi embolize to the cerebral circulation, leading to severe cerebral infarction. Strokes related to AF are associated with higher mortality and more severe neurologic disability [3–6].

Oxidized low density lipoprotein (Ox-LDL) has an important role in the pathogenesis of vascular atherosclerosis. It plays a pro-inflammatory and pro-atherogenic role by inducing endothelial dysfunction [7]. Atherosclerotic thrombosis and oxidative stress play key roles in acute ischemic stroke (AIS) [8, 9]. Previous studies had shown that elevated plasma Ox-LDL levels were associated with coronary heart disease [10, 11]. High concentrations of plasma Ox-LDL were associated with an increased incidence of metabolic syndrome [12]. However, data from studies on the predictive value of Ox-LDL levels for outcomes of ischemic stroke were scarce.

Increasing evidence suggested that statins had pleiotropic effects in addition to a lipid-lowering effect [13, 14]. In clinical trials, statins had been shown to reduce cardiovascular events, including stroke, myocardial infarction and death [15–17]. Early statins administration enhances thrombolysis, augments antithrombotic responses, modulates endothelial nitric oxide synthase (eNOS), reduces nitric oxide production, decreases matrix metalloproteinase-9 (MMP-9) and ROS levels, increases cerebral blood flow [18–20]. On the other hand, statins could attenuate thrombus formation and thrombus growth, reduce infarct volume [16, 17].

In a previous study, poststroke statins use was associated with improved 5-year survival in patients with AF-related stroke [21]. However, poststroke assessment of statins use was susceptible to some interference, such as survivor bias. A largest meta-analysis showed that statins use at the time of stroke onset was associated with improved clinical outcomes [22], but there was no AF subgroup analysis. There were limited studies about the potential use and effectiveness of reduce the plasma Ox-LDL and improve the outcome of AF-related stroke.

In this study, we assembled a cohort of patients with AF-related AIS from four centers. The purpose was to investigate whether prestroke statins use can reduce plasma Ox-LDL levels and improve 3-month clinical outcomes in patients with AF-related AIS.

## Methods

### Study population

This study was a multicenter prospective study conducted in four medical centers: The Second People’s Hospital of Chengdu, Nuclear Industry 416 Hospital, Yongchuan Hospital and the First Affiliated Hospital, Chongqing Medical University. Patients with AIS were consecutively admitted to the stroke unit within 72 h of symptom onset from October 2015 to April 2018. Stroke was diagnosed according to clinical presentation, neurologic examination (sudden neurological deficit > 24 h that has a presumable vascular etiology), and the results of brain computed tomography (CT) scan or diffusion-weighted imaging magnetic resonance imaging (MRI). Electrocardiogram evidence of AF was confirmed in all patients within the prior 6 months. Ischemic stroke was not explained by other etiologies, such as large artery atherosclerosis, tumor, infection, vasculitis, small vessel disease, or procedural or surgical complication. Etiology of stroke was classified according the modified Trial of ORG 10172 in Acute Stroke Treatment (TOAST) criteria in four medical centers. AF-relate stroke was confirmed by an experienced neurologist who blinded to the study. The severity of stroke was assessed using the National Institutes of Health Stroke Scale (NIHSS).

### Inclusion criteria

Patients were included in the study only if they fulfilled all the following criteria: 1. Admission for first-ever AIS within 72 h, and modified Rankin Scale (mRS) score was 0 before AIS; 2. Evidence of acute ischemic lesion consistent with clinical manifestations, and sudden neurological deficit > 24 h; 3. No patients received thrombolytic and mechanical thrombectomy therapy (because those patients undergo a different therapeutic strategy and a high percentage of hemorrhagic transformation); 4. Patients had to have electrocardiogram-confirmed AF within the prior 6 months. The CHA2DS2-VASc scoring system assesses the risk of stroke, with a score of 2 or greater indicating a need for anticoagulation. All patients received standard treatment after admission, which consisted of antiplatelet or anticoagulant therapy, lipid-lowering medications and so on.

### Exclusion criteria

Exclusion criteria included 1. A history of acute myocardial infarction, rheumatic heart disease, valvular atrial fibrillation, other severe arrhythmias (other severe atrioventricular block and supraventricular arrhythmia); 2. Vasculitis, renal failure (estimated glomerular filtration rate < 30 ml/min.1.73 m^2^), active malignancies, severe pulmonary disease, hypohepatia, or procedural or surgical complications; and 3. A history of deep venous thrombosis or pulmonary embolism; 4. Acute or chronic systemic inflammatory disease or autoimmune diseases. 5. Cerebral hemorrhage, fever (≥38 °C), or hypoxia (arterial oxyhemoglobin saturation < 90%) on admission.

### Data collection

The previous medical history of all patients was collected to confirm whether statins were used before stroke. To rule out other severe arrhythmias and determine AF type, all patients underwent a 72 h-Holter electrocardiogram monitoring on the next day after admission, which was evaluated by two investigators who were blinded for patients. The interobserver data differences were resolved by consensus. Cardiac ultrasonography examination was performed on admission to exclude rheumatic heart disease.

Blood samples were collected from all stroke patients on admission and at 3 months. Plasma Ox-LDL levels were measured using the enzyme-linked immunosorbent assay (ELISA) (E-EL-H0124c, Wuhan, China) according to manufacturer’s protocol. Each sample was assayed in duplicate. The intraassay variation among the duplicates for all samples was < 10%. The Ox-LDL levels were expressed in U/L. Level of plasma Ox-LDL was considered abnormal if it was ≥3.4u/l.

All patients were followed up once a month for a total of 3 months. All patients were followed up by face-to-face interview, and patient’s information was obtained from hospital medical records. Patients were evaluated by trained staffs, who were blinded to patients’ baseline clinical status. The outcome was defined as death, major disability (scores 3–5 on the modified Rankin Scale [mRS]), and composite outcome (death/major disability) at 3 months after stroke onset.

### Statistical analysis

First, patients were classified into prestroke statins use and no prestroke statins use groups according to statins use before stroke on admission. The data are presented as median values (interquartile range [IQR]), numbers (%), or mean values (±standard deviation). Demographic characteristics, vascular risk factors, current smoking, and so on were compared between the 2 subgroups by univariate analysis using Pearson χ2 test, Fisher exact 2-sided test, or Student’s t test, distributions of continuous variables were determined by the Kolmogorov–Smirnov test, Mann–Whitney two sample test was applied in case of non-normal distributions. Second, we performed logistic regression analyses to determine the association between baseline plasma Ox-LDL levels,3-month plasma Ox-LDL levels, prestroke statins use and outcomes (death, major disability and death/major disability), adjusting for all confounders (age, baseline NIHSS score, mean AF duration, CHA2DS2-VASc score, sex, BMI, hypertension, current smoking, current alcohol consumption, diabetes, hyperlipidemia, AF type, family history of stroke, prestroke statins use, Ox-LDL levels and use of antihypertensive, warfarin, direct oral anticoagulants, antiplatelets). The results were expressed as adjusted odds ratios (OR) with their corresponding 95% confidence intervals (CI). The data were analyzed using SPSS 22 software. *P* < 0.05 was considered statistically significant.

## Results

### Characteristics of the patients

A total of 242 AIS patients with AF (118 men; 124 females) were included in this study, their mean AF duration was 67.7 months. Of all patients, 85 had paroxysmal AF and 157 had persistent AF. 57 (23.6%) patients received anticoagulants, and 40(16.5%) patients received antiplatelets. 106 (43.8%) were assigned to the prestroke statins use group. The baseline characteristics of patients in the no prestroke statins use group (136) and prestroke statins use group (106) were compared (Table 1). At baseline, 169 patients had hypertension, 134 patients were received antihypertensive therapy, there was no difference between two group(*P* > 0.005). Patients with prestroke statins use showed a significantly higher prevalence of hyperlipidemia than that in patients with no prestroke statins use (*P* < 0.001). Patients with statins use showed significantly lower NIHSS score and Ox-LDL levels than patients with no statins use (*P* < 0.005).

**Table 1:** Comparison of baseline characteristics between patients with no prestroke statins use prestroke statins use groups

	No prestroke statins use group (136)	Prestroke Statins use group (106)	OR(95% CI)	P*
Age, y (Mean SD)	66.1 ± 10.5	67.2 ± 9.9		0.303
NIHSS score, median (IQR)	11 (7–14)	8(6–12)		**0.041**
Mean AF duration, m (Mean SD)	67.7 ± 10.0	67.8 ± 11.3		0.915
CHA2DS2-VASc, median (IQR)	4(3–5)	4(3–5)		0.090
Females, n (%)	66 (48.5%)	58 (54.7%)	1.29 (0.77–2.13)	0.339
Men, n (%)	70 (51.5%	48 (45.3%)	1.29 (0.77–2.13)	0.339
BMI ≥ 24 kg/m, n (%)	36 (26.5%)	37 (34.9%)	1.50 (0.86–2.59)	0.156
Hypertension, patients, n (%)	90 (66.8%)	79 (74.5%)	1.50 (0.85–2.63)	0.160
Current Smoking, n (%)	46 (33.8%)	30 (28.3%)	0.77 (0.45–1.34)	0.359
Current alcohol consumption, n (%)	41 (30.2%)	24 (22.6%)	0.67 (0.38–1.22)	0.191
Diabetes, n (%)	37 (27.2%)	31 (29.2%)	1.11 (0.63–1.94)	0.736
Hyperlipidemia, n (%)	40 (29.4%)	79 (74.5%)	7.02 (3.96–12.4)	**< 0.001**
AF Type				
Paroxysmal, n (%)	47(34.6)	38(35.8)	0.95(0.56–1.61)	0.835
Permanent, n (%)	89(65.4)	68(64.2)	0.95(0.56–1.61)	0.835
Family history of stroke, n (%)	28 (20.6%)	23 (21.7%)	1.07 (0.57–1.99)	0.834
Ox-LDL,U/L (Mean SD)	37.6 ± 5.1	33.5 ± 5.5		**< 0.001**
Medication use				
Warfarin, n (%)	20 (14.7%)	19 (17.9%)	1.27 (0.64–2.52)	0.499
direct oral anticoagulants, n (%)	10(7.4%)	8(7.5%)	1.03(0.39–2.70)	0.954
antiplatelets, n (%)	25(18.4)	15(14.2)	0.73(0.36–1.45)	0.379
Antihypertensive, n (%)	76 (55.9%)	58 ((54.7%)	0.95 (0.57–1.59)	0.856

Bold indicates *P*-values less than 0.05

*Comparison between no prestroke statins use and prestroke statins use groups. The data are presented as median values (interquartile range [IQR]), numbers (%), or mean values (±standard deviation). Categorical variables are expressed as frequency (percent) for *P* values. Baseline characteristics were compared between the 2 subgroups by univariate analysis using Pearson χ2, distributions of continuous variables were determined by the Kolmogorov–Smirnov test, Mann–Whitney two sample test was applied in case of non-normal distributions

At discharge, all the patients were prescribed statins; 110 patients were prescribed direct oral anticoagulants, 61 in no prestroke statins group and 49 in prestroke statins group; 85 patients were prescribed warfarin, 46 in no prestroke statins group and 39 in prestroke statins group; 35 patients were prescribed antiplatelets, 25 in no prestroke statins group and 15 in prestroke statins group. 7 patients had intracranial hemorrhage conversion and 5 patients had gastrointestinal bleeding, who did not receive the anticoagulants and antiplatalets at discharge. After 4–8 weeks, 5 patients were prescribed direct oral anticoagulant, and 7 patients were prescribed antiplatelet therapy. No bleeding complications occurred during the follow-up period, and no patients stopped taking anticoagulants or antiplatelet drugs. There was no difference in direct oral anticoagulants, warfarin and antiplatelets between two groups (*P* > 0.05). During the 3-month follow-up period, no patients were denied follow-up, and no patients had adverse reactions of statins.

### Plasma ox-LDL levels in the prestroke statins use and no prestroke statins use groups

Plasma Ox-LDL levels were significantly lower in the prestroke statins use group on admission (33.5 ± 5.5 vs 37.6 ± 5.1, *P* < 0.001). Compared with the baseline, Ox-LDL levels of the 3-month treatment period decreased both in two groups, the prestroke statins use group had lower Ox-LDL level (24.4 ± 7.6 vs 29.3 ± 6.0, *P* < 0.001). Ox-LDL levels decreased about 27.1% in the prestroke statins use group, 22.1% in the no prestroke statin use group. The data showed significantly different Ox-LDL levels in the two groups at different time points.

### Association between plasma ox-LDL levels and prognosis

We also analyzed whether the plasma Ox-LDL levels had effect on outcomes. 70 (70/242, 28.9%) patients had died during follow-up, and 86 patients had major disability at 3 months. Compared with the surviving patients, the patients who died had higher Ox-LDL levels (37.9 ± 5.6 vs 35.0 ± 5.5, *P* < 0.001) on admission, and at 3 months (28.8 ± 4.7 vs 26.5 ± 7.3, *P* = 0.039). Patients with major disability had higher Ox-LDL levels than patients with good prognosis on admission (36.4 ± 6.0vs 33.6 ± 4.6, *P* = 0.004), there was no significant difference between two groups at 3 months (27.6 ± 6.4 vs 25.4 ± 8.0, *P* = 0.099). In the multivariable logistic regression model, adjusting for age, baseline NIHSS score, mean AF duration, CHA2DS2-VASc score, sex, BMI, hypertension, current smoking, current alcohol consumption, diabetes, hyperlipidemia, AF type, family history of stroke, prestroke statins use, and use of antihypertensive, warfarin, direct oral anticoagulants, antiplatelets, baseline plasma Ox-LDL levels were associated with increased risk of 3-month mortality (adjusted OR, 1.05; 95% CI, 0.99–1.12; *P* = 0.047), there was no association between baseline plasma Ox-LDL levels and major disability (adjusted OR, 1.04; 95% CI, 0.96–1.12; *P* = 0.335), and composite outcome (adjusted OR, 1.07; 95% CI, 0.99–1.14; *P* = 0.072); similarly, 3-month plasma Ox-LDL levels were not associated with mortality (adjusted OR, 1.05; 95% CI, 0.99–1.10; *P* = 0.096), major disability (adjusted OR, 1.04; 95% CI, 0.99–1.10; *P* = 0.182), and composite outcome (adjusted OR, 1.04; 95% CI, 0.98–1.09; *P* = 0.146).

### Multivariable models on the association between prestroke statins use and mortality, major disability, and composite outcome

Patients who died had a significantly higher NIHSS score (13.4 ± 6.4 vs 9.1 ± 4.1, *P* < 0.001), older age (68.6 ± 11.3 vs 65.7 ± 9.7, *P* = 0.022), and lower percentage of prestroke statins use [32.9% (23/70) vs 48.3% (83/172); OR, 0.53 (95% CI, 0.30–0.94); *P* = 0.029] on admission.

Among the survivors, 32 (32/83, 38.6%) patients had a major disability in the prestroke statins use group, which was lower percentage than that in patients with no prestroke statins use (54/89, 60.7%); patients with 3-month major disability had a significantly higher NIHSS at admission (10.3 ± 4.2 vs 8.0 ± 3.7, *P* < 0.001) than that in patients with a good prognosis (Table 2).

**Table 2:** Prognosis between patients with no statin and statin groups

	No prestroke statins use group (136)	No prestroke statins use group (106)	OR (95% CI)	P*
Death, n (%)	47 (34.6)	23 (21.7)	0.53 (0.29–0.94)	0.029
Major disability, n (%)	54/89 (60.7)	32/83 (38.6)	0.41 (0.22–0.75)	0.004
Major disability+death, n (%)	101 (74.3)	55 (51.9)	0.37 (0.22–0.64)	< 0.001

*Comparison between no pre-stroke statins use group and pre-stroke statins use group. Categorical variables are expressed as frequency (percent) for *P* values, prognosis was compared between the 2 subgroups by univariate analysis using Pearson χ2 test

In the multivariable logistic regression model, after adjusting for all confounders (Model 1), prestroke statins use was associated with decreased risk of 3-month mortality (*P* = 0.031), major disability (*P* = 0.047) and composite outcome (*P* = 0.009), and higher NIHSS score was associated with increased risk of 3-month mortality (*P* = 0.000), major disability (*P* = 0.003) and composite outcome (*P* < 0.001); even when 3-month plasma Ox-LDL levels were entered into multivariate logistic regression (Model 2), prestroke statins use resulted to be associated with decreased risk of 3-month mortality (*P* = 0.023), major disability (*P* = 0.038) and composite outcome (*P* = 0.003), and higher NIHSS score was associated with increased risk of 3-month mortality (*P* < 0.001), major disability (adjusted OR, *P* = 0.003) and composite outcome (*P* < 0.001) (Table 3).

**Table 3:** Multivariable models showing association between prestroke statins use, NIHSS and Prognosis

	OR (95% CI)	P*
Model 1 (baseline Ox-LDL)		
Prestroke Statins use		
Mortality	0.38(0.16–0.91)	**0.031**
Major disability (mRs3–5)	0.38 (0.15–0.99)	**0.047**
Major disability (mRs3–5) + death	0.31(0.17–0.74)	**0.009**
NIHSS score		
Mortality	1.18(1.10–1.26)	**< 0.001**
Major disability (mRs3–5)	1.17(1.06–1.30)	**0.003**
Major disability (mRs3–5) + death	1.20(1.10–1.29)	**< 0.001**
Model 2(3-month Ox-LDL levels)		
Prestroke Statins use		
Mortality	0.36(1.15–0.87)	**0.023**
Major disability (mRs3–5)	0.38(0.15–0.95)	**0.038**
Major disability (mRs3–5) + death	0.27(0.11–0.64)	**0.003**
NIHSS score		
Mortality	1.18(1.11–1.27)	**< 0.001**
Major disability (mRs3–5)	1.17(1.06–1.30)	**0.003**
Major disability (mRs3–5) + death	1.19(0.10–1.29)	**< 0.001**

Bold indicates P-values less than 0.05

*Multivariable adjusted for age, baseline NIHSS score, mean AF duration, CHA2DS2-VASc score, sex, BMI, hypertension, current smoking, current alcohol consumption, diabetes, hyperlipidemia, AF type, family history of stroke, prestroke statins use, baseline Ox-LDL levels (3-month Ox-LDL levels) and use of antihypertensive, warfarin, direct oral anticoagulants, antiplatelets

## Discussion

In this study, 242 patients were included,79.3% patients with hypertension were received antihypertensive therapy, 89.1% patients with were received statins therapy, only 23.6% patients were received anticoagulation. Previous studies had shown that the treatment rate of patients with hypertension, hyperlipidemia, and AF was about 30.1, 20.0% respectively [23, 24], the proportion of hypertension and hyperlipidemia, who were receiving treatment varied significantly across subpopulations; which were associated with male sex, age, income, and an absence of previous cardiovascular events, diabetes, obesity, or alcohol use. In our study, most of the patients came from cities, the treatment rate of hypertension and hyperlipidemia was higher than that the findings of previous studies [24, 25].

AF can increase platelet reactivity, lead to thrombin generation, and platelet aggregation, which underlie the formation of a thrombus, and the size of a thrombus can affect the infarct volume and severity of stroke [25, 26]. According to previous study, AF increases the incidence of stroke fivefold, patients with cardioembolic strokes are at greater risk of death, severe disability and risk of hemorrhagic transformation [27]. Oral anticoagulants are the most effective treatment for preventing cardioembolic stroke, but in our country, the rate of AF treatment is very low. In our study, all included patients have electrocardiogram-confirmed AF within the prior 6 months, and no patients were newly diagnosed with AF after stroke,57(23.56%) patients were receiving oral anticoagulants pior to the index stroke, and this is consistent with the findings of previous studies [28]. The results suggesting that better education and awareness are needed to improve efforts for stroke prevention among AF patients.

Statins had pleiotropic effects in addition to LDL-C lowering. Data had shown that statins had benefits on the endothelium, platelets, myocardium. Severe studies showed that prestroke statins use was associated with milder stroke severity at stroke onset [29, 30]. In this study, most of the patients who used statins before stroke had hyperlipidemia. At baseline, patients with prestroke statins use showed a significantly higher prevalence of hyperlipidemia than that in patients with no prestroke statins use. We found that prestroke statins use among AF-related stroke was associated with lower NIHSS score on admission, this study suggested that the prestroke statins use might reduce the severity of stroke. Several studies had demonstrated a beneficial effect of baseline statins on the outcome of patients with stroke [31, 32]; patients with prestroke statins were more likely to achieve good functional outcome, but few studies had focused on stroke associated with AF. Examining AF patients was important, because the source of these strokes is the left atrium or left atrial appendage, and AF-related stroke is associated with high morbidity and mortality. A previous analysis of lipid lowering treatment in the Atrial Fibrillation Follow-up Investigation of Rhythm Management (AFFIRM) trial demonstrated decreased ischemic stroke (HR 0.56;95% CI,0.36 to 0.89; *P* = 0.01) among individuals with AF used lipid lowering therapy at baseline, but this analysis did not evaluate differences in ischemic stroke functional outcomes. A meta-analysis showed that the association of prestroke statins use and good functional outcome was significant in patients with large artery atherosclerosis and small vessel occlusion, but not in cardioembolic stroke [33]. In a recent study by Ko et al., that enrolled 1030 AF-related ischemic stroke patients followed up for 30 days, the authors aimed to observe the influence of prestroke statins use on functional outcome; the results showed that prestroke statins use among patients with AF-related ischemic stroke was associated with a 32% reduction in the risk of the stroke being severe at the time of discharge or decreased mortality at 30 days [34], but the limitation of this study was that the patients were followed up for a short time, only investigated clinical outcome at discharge and the 30-day mortality rate. In our study, all the patients were followed for 3 months, we found that prestroke statins use was independently associated with lower mortality and major disability in AF-related AIS.

There are biological mechanisms underlying the association between statins use and improved clinical outcomes after ischemic stroke. Prestroke statins use was associated with increased collateral blood flow, which might be beneficial in cardioembolic stroke and reduce infarct size [35, 36], and in the animal model, atorvastatin use could increase angiogenic responsiveness [31, 37], and rosuvastatin could increase neovascularization via mechanisms independent of lipid lowering [32]. Statins could reduce platelet activation, inhibit subclinical inflammation, oxidative stress, improve endothelial dysfunction [38–42], and significantly reduce venous thromboembolism [42]. Statins had also been reported to decrease levels of C-reactive protein, interleukin-6, TNF-α, improve left ventricular function among individuals with heart failure [43].

The mechanism of increased plasma Ox-LDL levels after AIS is unclear, although it has been speculated that AIS is associated with enhanced oxidative stress, which can further oxidize native LDL-cholesterol to Ox-LDL [44, 45]. Another possible explanation might be the increase in lipolysis and lipid peroxidation after AIS [46, 47]. In this study, we found that plasma Ox-LDL levels were high on admission and decreased at 3 months after stroke. We also found that Ox-LDL levels were significantly lower in the prestroke statins use group on admission, and elevated plasma Ox-LDL levels were independently associated with increased risk of 3-month mortality. These findings suggest that plasma Ox-LDL levels may be a useful marker for monitoring the oxidative status of the brain after cerebral infarction, and prestroke statins use might inhibit Ox-LDL production and protect endothelial function.

In this study, we found that prestroke statins use in patients presenting with AF-related ischemic stroke was associated with a reduction in the risk of major disability and death, this association persisted after adjustment for all confounders including age, baseline NIHSS score, mean AF duration, CHA2DS2-VASc score, sex, BMI, hypertension, current smoking, current alcohol consumption, diabetes, hyperlipidemia, AF type, family history of stroke, prestroke statins use, baseline or 3-month Ox-LDL levels and use of antihypertensive, warfarin, direct oral anticoagulants, antiplatelets, these results suggested that prestroke statins use could improve clinical outcome in AF-related AIS.

Some limitations of this study merit consideration. First, in most cases we relied on baseline and 3-month blood sample, thus we could not account for variations in Ox-LDL levels that occur over time. Repeat assays could provide additional information on its prognostic implications in AF-related AIS. Second, the limitations of our study were related to the small number of patients and the short duration of the study. Third, although we adjusted for NIHSS score, which had been shown to correlate with infarction volume, we lacked data on infarction volume. Forth, there were many different types of statins, and it was possible that each drug had different effects on the prognosis of stroke, this study did not separately list the effects of a given statin on the prognosis of stroke. Furthermore, we did not know the exact time and dose of statins, so the relationship between dose and time dependence on prognosis had not been assessed. Fifth, patients receiving thrombolytic and mechanical thrombectomy therapy were excluded, which limits the generalizability of the results. Sixth, Ox-LDL has an established role in the pathogenesis of atherosclerosis, but the carotid stenosis or vessel occlusion were not analyzed in this study, which might have an impact on the results. Seventh, we could not assess recurrent vascular events but only disability and mortality. The strength of our study are that it was a multicenter clinical study the collection of several important clinical data, the adjustment of data analyses for a wide variety of confounding factors.

## Conclusions

In conclusion, our findings indicated that prestroke statins use could reduce plasma Ox-LDL levels and improve clinical outcomes in patients with AF-related AIS.

## Data Availability

The datasets used and/or analyzed during the current study are available from the corresponding author on reasonable request.

## Abbreviations

AF: Atrial fibrillation
AIS: Acute ischemic stroke
CI: Confidence Interval
IQR: Interquartile range
M: Mean
OR: Odds Ratio
Ox-LDL: Oxidized low density lipoprotein
SD: Standard Deviation
